# 

**Published:** 1882-06

**Authors:** 


ESTABLISHED IN 1855.

OLD SERIES. Vol. XX—No. 3. | JUNE, 1882. | NEW SERIES Vol. I—No 9

S.G.O. INDEXED

### ATLANTA

### MEDICAL REGISTER.

*(ATLANTA MEDICAL AND SURGICAL JOURNAL.)*

EDITED BY

JAMES B. BAIRD, M.D.

H. H. DICKSON, PUBLISHER. TERMS, $2.50 A YEAR IN ADVANCE

ATLANTA, GEORGIA:

*H. H. DICKSON, BOOK AND JOB PRINTER AND BOOKBINDER.*

1882.

